# Prediction of Endometrial Carcinoma Using the Combination of Electronic Health Records and an Ensemble Machine Learning Method

**DOI:** 10.3389/fmed.2022.851890

**Published:** 2022-03-04

**Authors:** Wenwen Wang, Yang Xu, Suzhen Yuan, Zhiying Li, Xin Zhu, Qin Zhou, Wenfeng Shen, Shixuan Wang

**Affiliations:** ^1^Department of Obstetrics and Gynecology, Tongji Medical College, Tongji Hospital, Huazhong University of Science and Technology, Wuhan, China; ^2^School of Computer Engineering and Science, Shanghai University, Shanghai, China; ^3^Department of Obstetrics and Gynecology, Affiliated Renhe Hospital of China Three Gorges University, Yichang, China; ^4^Biomedical Information Engineering Lab, The University of Aizu, Fukushima, Japan

**Keywords:** machine learning, ensemble method, prediction, endometrial carcinoma (EC), model

## Abstract

Endometrial carcinoma (EC) is a common cause of cancer death in women, and having an early accurate prediction model to identify this disease is crucial. The aim of this study was to develop a new machine learning (ML) model-based diagnostic prediction model for EC. We collected data from consecutive patients between November 2012 and January 2021 at tertiary hospitals in central China. Inclusion criteria included women undergoing endometrial biopsy, dilation and curettage, or hysterectomy. A total of 9 features, including patient demographics, vital signs, and laboratory and ultrasound results, were selected in the final analysis. This new model was combined with three top optimal ML methods, namely, logistic regression, gradient-boosted decision tree, and random forest. A total of 1,922 patients were eligible for final analysis and modeling. The ensemble model, called TJHPEC, was validated in an internal validation cohort and two external validation cohorts. The results showed that the AUC values were 0.9346, 0.8341, and 0.8649 for the prediction of total EC and 0.9347, 0.8073, and 0.871 for prediction of stage I EC. Nine clinical features were confirmed to be highly related to the prediction of EC in TJHPEC. In conclusion, our new model may be accurate for identifying EC, especially in the early stage, in the general population of central China.

## Introduction

Globally, cancer of the corpus uteri (typically referred to as endometrial carcinoma, EC) ranked as the 6th most common type of cancer and 14th main cause of cancer death in women and caused an estimated 320,000 new cases and 76,000 deaths in 2012 (1). In China, the age-standardized incidence of EC had a significant upward trend in the period of 2000–2011 (2). Data from the China-Global Cancer Observatory has shown that EC ranked 10th for the incidence and mortality of cancer and caused 73,253 new cases and 13,329 deaths in 2018 (WHO CHINA CANCER 2018). The poor prognosis of EC was closely connected with the higher stage. The 5-year relative survival rates of EC decreased by 79% with staging from 95% for stage I to 16% for stage IV (3).

To improve the survival rates for women with EC, researchers have developed a series of prediction models to facilitate the early diagnosis of symptomatic patients, especially those with postmenopausal bleeding (4, 5). Current data provide some advice for the application of limiting transvaginal sonography screening for higher-risk groups identified according to epidemiological risk factors. This combination has improved the diagnostic sensitivity and specificity to 84 and 90%, respectively (5). However, this well-established model can be implemented only in postmenopausal women.

The application of big data to medicine has prompted recent advances in data analytics. The application of artificial intelligence in diagnostics will help doctors improve accuracy in various fields such as medical imaging, bioinformatics, and electronic health records (EHRs) (6–9). Recently, Pergialiotis et al. used artificial neural networks (NNs), and classification and regression trees for the prediction of EC in postmenopausal women in Greece. Compared with traditional statistical analysis (classical regression analysis), the artificial NNs had superior predictive accuracy, with a sensitivity of 86.8% and specificity of 83.3% (10). However, the restricted population and lack of interpretability have led to the limited use of this model. Hart et al. used random forest (RF) and NN models based only on personal health records for predicting EC risk and stratifying the population included in the Prostate, Lung, Colorectal, and Ovarian Cancer Screening Trial. This trial enrolled participants 55–75 years of age between November 1993 and July 2001, and the AUC values of these models in the study by Hart et al. were 0.96 and 0.91, respectively (11). However, the narrow data and the restricted population involved in the models confined its positive predictive value and application. And Troisi et al. had combined a serum metabolomic signature of endometrial carcinoma with an ensemble machine learning algorithm for endometrial cancer screening with >99% accuracy lately (12). However, this accurate predictive model just focused on postmenopausal women.

To fill the gap applications of these models, we aimed to develop a new computer-assisted predictive model for the early detection of EC that may assist in clinical decision-making for the general population of China.

## Methods

### Study Population

Our retrospective research was conducted in the Department of Obstetrics and Gynecology at Tongji Hospital, Tongji Medical College, Huazhong University of Science and Technology (TJH), and the Department of Obstetrics and Gynecology at the affiliated Renhe Hospital of China Three Gorges University (RHH). Data from consecutive selected patients were collected between November 2012 and January 2021. Inclusion criteria included women undergoing endometrial biopsy, dilation and curettage, or hysterectomy. Women with endometrial atypia hyperplasia, any prior malignancy, current pregnancy, or severe infectious disease were excluded. This study was approved by the Medical Ethics Committee of Tongji Hospital Affiliated to Tongji Medical College of Huazhong University of Science and Technology and filed in the Medical Ethics Committee of the affiliated Renhe Hospital of China Three Gorges University-as a sub-center of this study.

### Study Definitions

According to the endometrial pathology diagnoses, patients were classified into two categories: the case group with EC and the control group without EC (referred as benign group). The benign group consisted of women with a normal endometrium, endometrial polyps, hyperplasia without atypia, or submucosal uterine fibroids. The case group included women with endometrioid adenocarcinoma or other types such as clear cell carcinoma of the endometrium, endometrial stromal sarcoma, serous carcinoma, carcinosarcoma, or large- or small-cell neuroendocrine carcinoma. The details are shown in Supplementary Table 1.

### Data Preprocessing

**Figure 1:**
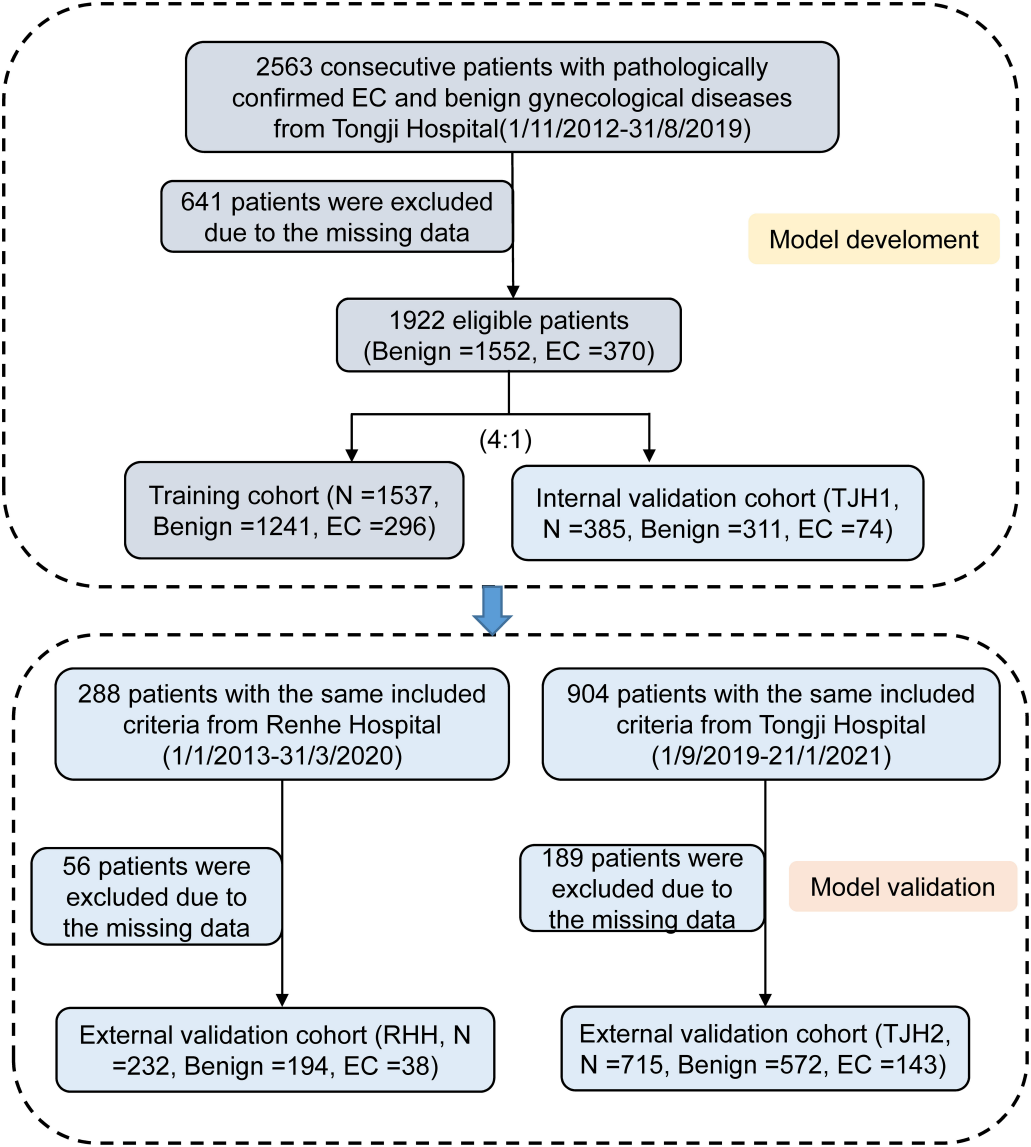
Flow chart of group allocation. EC, endometrial carcinoma.

**Figure 2:**
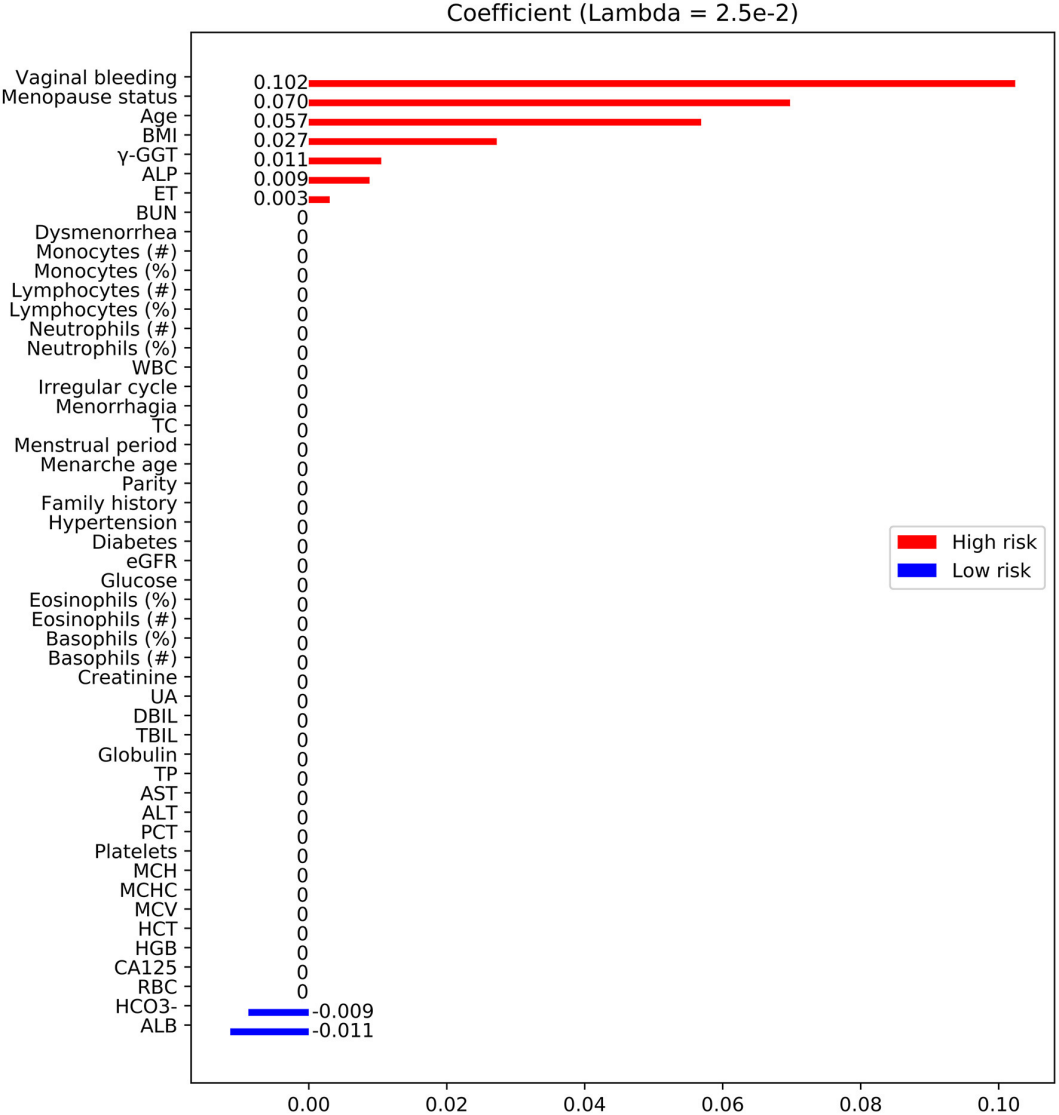
Feature selection using LASSO. Feature coefficients revealed high-risk (red bar) and low-risk (blue bar) features, which were selected using LASSO with an optimal lambda value of 2.5 × 10^−3^. BMI, body mass index; TBIL, total bilirubin; ALP, alkaline phosphatase; BUN, blood urea nitrogen; γ-GGT, γ-glutamyl transpeptidase; ET, endometrial thickness; TC, total cholesterol; AST, aspartate aminotransferase; CA-125, cancer antigen 125; UA, uric acid; Diabetes, type 2 diabetes; Family history, family history of malignant diseases; WBC, white blood cell; RBC, red blood cell; ALT, alanine aminotransferase; HGB, hemoglobin; PCT, thrombocytocrit; MCH, mean corpuscular hemoglobin; MCV, mean corpuscular volume; HCT, hematocrit; eGFR, estimated glomerular filtration rate; MCHC, mean corpuscular hemoglobin concentration; TP, total protein; DBIL, direct bilirubin; ALB, albumin; ${\text{HCO}}_{3}^{\text{-}}$, concentrations of bicarbonate.

A total of 3,755 consecutive patients with EC or benign gynecological disease were enrolled. Patients were removed if the incomplete data accounted for >20% of those used in the model development and internal validation cohort. Fifty-two features relating to clinical characteristics, laboratory test results, and ultrasound data were extracted from EHRs. About 23% of cases were deleted because of missing data (Figure 1). Utility of predictive mean matching was conducted using R version 4.0.2 (R Development Core Team, Vienna, Austria; https://www.R-project.org/) to fill in missing data. The feature selection was conducted using least absolute shrinkage and selection operator (LASSO) regression. Nine features were introduced into the final analytic cohort (Figure 2). The definition of variables was shown in Supplementary Table 6.

### Model Development

The data from TJH obtained between November 2012 and August 2019 were randomly divided into two cohorts—the training and internal validation cohorts—at a ratio of 4:1, respectively. The data from RHH and from TJH obtained between September 2019 and January 2021 were used as two external validation cohorts (termed here the RHH cohort and TJH2 cohort, respectively). The training cohort was analyzed using six machine learning (ML) models, namely, logistic regression (LR), support vector machine (SVM), K-nearest neighbor (KNN), RF, gradient-boosted decision tree (GBDT), and NN. Five-fold cross-validation was applied to fine-tune the parameters of these models (Supplementary Figure 2). Ultimately, an ensemble model, named TJHPEC, was generated from the three top-ranked predictive models (LR, RF, and GBDT). Specifically, the incidence risk probability of each estimator (LR, RF, and GBDT) was integrated by manually assigning weights of 0.2, 0.2, and 0.6, respectively. The well-fitted models were then tested using the test cohort. A normalized probability of risk for EC ranging from 0 to 1 was produced by each model. We selected the threshold of 0.34 to assign the predicted incidence risk label by optimizing the F1 score for the training and validation cohorts. Probabilities of <0.34 were assigned to non-EC and otherwise to EC for all ML methods (Supplementary Figure 1). Python was used for model training and prediction. The LR, SVM, KNN, RF, GBDT, and NN models were called using the methods Logistic Regression, SVC, KNeighborsClassifier, RandomForestClassifier, Gradient BoostingClassifier, and MLPClassifier with prominent settings after five-fold cross-validation. To make the data more Gaussian-like, StandardScaler was used to standardize the features before training and prediction by subtracting the mean and scaling to unit variance.

### Data Analysis

The non-parametric Kruskal–Wallis test was used to analyze the clinical characteristics as continuous variables, and the chi-squared test for categorical variables. Multiple imputation with predictive mean matching was conducted to impute missing data for continuous variables. LR was applied as a traditional method for the prediction of EC. The predictive model was estimated using the receiver-operating characteristic (ROC) curve, and the accuracy, sensitivity, specificity, positive predictive value (PPV), negative predictive value (NPV), F1 score, Cohen's kappa, Brier score, and related 95% confidence intervals (CIs) were calculated. All statistical analyses were performed using R version 4.0.2 (R Development Core Team). LR and RF models were performed in the Python 3.7.0 (https://www.python.org/) environment. For all tests, *p* < 0.05 was considered to be significant. The schematic diagram of our predictive model was summarized in Supplementary Figure 2.

## Results

### Patients' Characteristics

To establish this diagnostic prediction model, 2,563 consecutive patients with pathologically confirmed EC and benign gynecological disease were enrolled at Tongji Hospital of Tongji Medical College, Huazhong University of Science and Technology, between November 2012 and August 2019. A total of 641 patients were excluded for missing data, and the remaining 1,922 patients had data that were eligible for analysis and modeling. The EHRs of 1,537 patients were randomly assigned to the training cohort, and the other 385 patients were assigned to the group for internal validation (TJH1 cohort). For external validation, the EHRs of 288 patients with roughly the same characteristics as the RHH cohort and 904 from the TJH2 cohort were considered. Of these cohorts, 56 patients in the first group were excluded because of missing data, and the remaining 232 patients were eligible; 189 patients in the TJH2 cohort were excluded, and the remaining 715 patients were eligible. The detailed flowchart and disease classification are shown in Figure 1 and Supplementary Table 1, respectively.

The demographics of the eligible patients included in the validation are presented in Table 1.

**Table 1:** Demographic characteristics in the validation cohorts.

**Characteristic (Mean ± SD) or *N* (%)**	**TJH1 cohort**		**RHH cohort**		**TJH2 cohort**	
	**Benign (*n* = 311)**	**EC (*n* = 74)**	**Benign (*n* = 194)**	**EC (*n* = 38)**	**Benign (*n* = 572)**	**EC (*n* = 143)**
Age, years	40 ± 11	54 ± 8	43 ± 10	54 ± 10	42 ± 11	54 ± 8
BMI, kg/m^2^	22.1 ± 3.3	24.5 ± 4.0	22.5 ± 3.0	24.3 ± 3.7	22.8 ± 3.4	24.9 ± 3.6
**Menstrual history**						
Menopause status	32 (10.3)	42 (56.8)	23 (11.9)	25 (65.8)	63 (11)	78 (54.5)
**Symptoms**						
Vaginal bleeding	108 (34.7)	68 (91.9)	106 (54.6)	32 (84.2)	227 (39.7)	128 (89.5)
**Laboratory tests**						
ALB, g/L	41.7 ± 4.4	38.8 ± 5.7	41.9 ± 6.0	40.7 ± 4.8	42.9 ± 4.2	41.8 ± 3.9
ALP, U/L	54 ± 19	65 ± 22	60 ± 20	78 ± 23	62 ± 26	71 ± 20
γ-GGT, U/L	18 ± 17	27 ± 27	17 ± 15	21 ± 13	20 ± 25	26 ± 23
${\text{HCO}}_{3}^{\text{-}}$, mmol/L	24.0 ± 2.3	23.7 ± 3.1	25.9 ± 2.3	27.2 ± 2.5	23.7 ± 2.2	23.7 ± 2.3
**Ultrasound**						
ET, mm	8.2 ± 4.7	8.1 ± 7.7	9.7 ± 4.3	14.9 ± 6.9	7.3 ± 4.4	6.1 ± 5.8

*BMI, body mass index; ET, endometrial thickness; CA-125, cancer antigen 125*.

### Selection of Features

Fifty features were extracted from the EHRs, and <20% of these had missing data (Table 1; Supplementary Table 2). Nine of the 50 features were selected by LASSO analysis for the final modeling (Figure 2), which yielded 7 features associated with a high risk of EC: vaginal bleeding, menopause status, age, body mass index (BMI), alkaline phosphatase, endometrial thickness (ET), γ-glutamyl transpeptidase (γ-GGT). The 2 features associated with a low risk of EC were concentrations of bicarbonate, albumin.

### Performance of Models in the Internal and External Validations

Initially, we adopted six ML models (LR, SVM, GBDT, NN, KNN, and RF) to predict EC in these cohorts (Table 2; Supplementary Table 3). The top three diagnostic–predictive models (RF, GBDT, and LR) were fused to create a new ensemble model, named TJHPEC. The decision-making threshold of 0.34 and the best achievable F1 score were derived to achieve the optimum classifier for the prediction of EC, and this showed the best predictive power among other models in our test cohorts (Table 2).

**Table 2:** Performance indices of the four predictive models for total EC in the validation cohorts.

	**TJHPEC**	**LR**	**RF**	**GBDT**
**Internal validation cohort (TJH1)**				
AUC (95% CI)	0.9346 (0.9108–0.9584)	0.9175 (0.8901–0.9449)	0.932 (0.9077–0.9563)	0.9335 (0.9095–0.9575)
Accuracy (95% CI)	91.17% (88.33–94.00%)	88.83% (85.68–91.98%)	89.61% (86.56–92.66%)	90.65% (87.74–93.56%)
Sensitivity (95% CI)	86.49% (78.70–94.28%)	79.73% (70.57–88.89%)	89.19% (82.11–96.26%)	86.49% (78.70–94.28%)
Specificity (95% CI)	92.28% (89.32–95.25%)	91.00% (87.82–94.18%)	89.71% (86.33–93.09%)	91.64% (88.56–94.72%)
PPV (95% CI)	72.73% (63.42–82.03%)	67.82% (58.00–77.63%)	67.35% (58.06–76.63%)	71.11% (61.75–80.48%)
NPV (95% CI)	96.63% (94.58–98.68%)	94.97% (92.48–97.45%)	97.21% (95.31–99.12%)	96.61% (94.55–98.68%)
F1	0.7901	0.7329	0.7674	0.7805
Kappa	0.7347	0.6629	0.7022	0.7218
Brier score	0.088	0.112	0.104	0.094
**External validation cohort (RHH)**				
AUC (95% CI)	0.8341 (0.777–0.8912)	0.831 (0.7732–0.8888)	0.8196 (0.7593–0.8799)	0.8265 (0.7677–0.8853)
Accuracy (95% CI)	81.03% (75.99–86.08%)	78.02% (72.69–83.35%)	77.16% (71.75–82.56%)	80.60% (75.52–85.69%)
Sensitivity (95% CI)	57.89% (42.20–73.59%)	60.53% (44.99–76.07%)	71.05% (56.63–85.47%)	60.53% (44.99–76.07%)
Specificity (95% CI)	85.57% (80.62–90.51%)	81.44% (75.97–86.91%)	78.35% (72.56–84.15%)	84.54% (79.45–89.62%)
PPV (95% CI)	44.00% (30.24–57.76%)	38.98% (26.54–51.43%)	39.13% (27.61–50.65%)	43.40% (30.05–56.74%)
NPV (95% CI)	91.21% (87.09–95.32%)	91.33% (87.14–95.52%)	93.25% (89.40–97.10%)	91.62% (87.56–95.68%)
F1	0.5	0.4742	0.5047	0.5055
Kappa	0.3857	0.3434	0.372	0.3889
Brier score	0.19	0.22	0.228	0.194
**External validation cohort (TJH2)**				
AUC (95% CI)	0.8649 (0.8377–0.8921)	0.8574 (0.8293–0.8855)	0.8544 (0.8259–0.8829)	0.8607 (0.833–0.8884)
Accuracy (95% CI)	80.98% (78.10–83.86%)	78.88% (75.89–81.87%)	79.30% (76.33–82.27%)	81.12% (78.25–83.99%)
Sensitivity (95% CI)	80.42% (73.92–86.92%)	81.82% (75.50–88.14%)	86.01% (80.33–91.70%)	81.12% (74.70–87.53%)
Specificity (95% CI)	81.12% (77.91–84.33%)	78.15% (74.76–81.53%)	77.62% (74.21–81.04%)	81.12% (77.91–84.33%)
PPV (95% CI)	51.57% (45.01–58.13%)	48.35% (42.05–54.64%)	49.00% (42.82–55.19%)	51.79% (45.24–58.33%)
NPV (95% CI)	94.31% (92.26–96.36%)	94.50% (92.45–96.56%)	95.69% (93.84–97.54%)	94.50% (92.48–96.52%)
F1	0.6284	0.6078	0.6244	0.6322
Kappa	0.5087	0.4761	0.4959	0.5133
Brier score	0.19	0.211	0.207	0.189

*GBDT, gradient-boosted decision tree; LR, logistic regression; RF, random forest; AUC, area under the receiver-operating characteristic curve; PPV, positive predictive value; NPV, negative predictive value; 95% CI, 95% confidence interval*.

The area under the ROC curve (AUC) for the TJHPEC was 0.9346 (95% CI 0.9108–0.9584) for the internal validation cohort (TJH1) and indicated near-perfect discriminative ability. For the RHH cohort, the AUC of the TJHPEC was 0.8341 (95% CI 0.777–0.8912). The AUC for the TJH2 cohort was 0.8649 (95% CI 0.8377–0.8921). The accuracy, sensitivity, and specificity were 91.17% (95% CI 88.33–94%), 86.49% (95% CI 78.7–94.28%), and 92.28% (95% CI 89.32–95.25%), respectively, in the TJH1 cohort; 81.03% (95% CI 75.99–86.08%), 57.89% (95% CI 42.2–73.59%), and 85.57% (95% CI 80.62–90.51%), respectively, in the RHH cohort; and 80.98% (95% CI 78.1–83.86%), 80.42% (95% CI 73.92–86.92%), and 81.12% (95% CI 77.91–84.33%), respectively, in the TJH2 cohort (Table 2).

**Figure 3:**
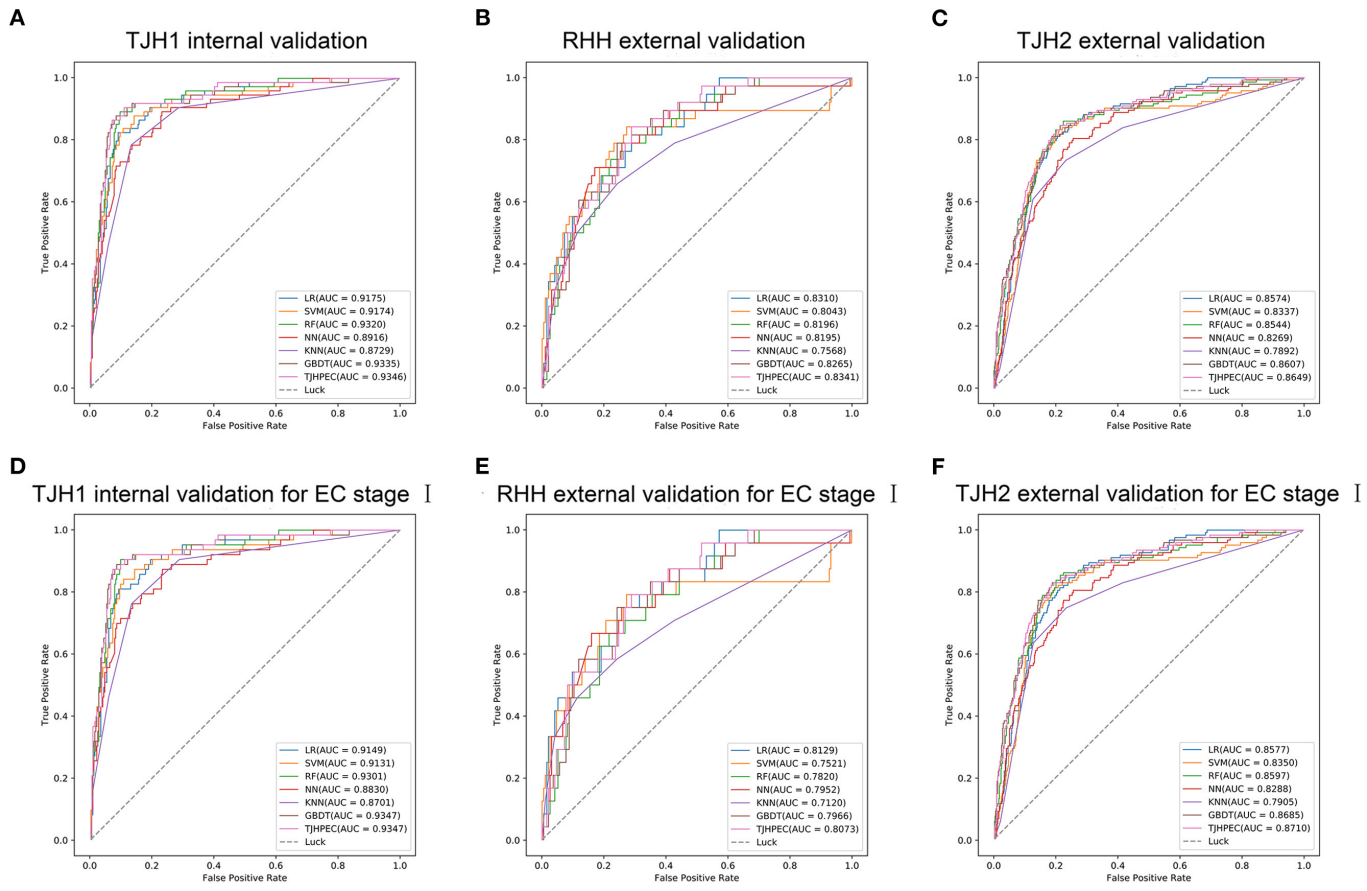
Evaluation of the efficiency of the diagnostic prediction of models (LR, SVM, RF, NN, KNN, GBDT, and TJHPEC) using ROC curves for the total EC cohorts (**A**, TJH1 internal validation set; **B**, RHH external validation set; **C**, TJH2 external validation set) and stage I EC cohorts (**D**, TJH1 internal validation set; **E**, RHH external validation set; **F**, TJH2 external validation set).

The Brier score is used to measure the accuracy of a probabilistic prediction. The Brier scores for the TJHPEC were 0.088, 0.19, and 0.19 for the TJH1, RHH, and TJH2 cohorts, respectively (Table 2). The parameters of predictive power in the three other models are shown in Supplementary Table 2. The ROC curves for the seven models (TJHPEC, RF, NN, GBDT, KNN, LR, and SVM) applied to the test cohort are shown in Figure 3.

ROC curves for the TJHPEC model for identifying stage I EC were created according to the International Federation of Gynecology and Obstetrics system and are shown in Figure 3 and Table 3. The AUC values were 0.9347 (95% CI 0.9102–0.9592), 0.8073 (95% CI 0.7335–0.8811), and 0.871 (95% CI 0.8435–0.8985) for the TJH1, RHH, and TJH2 cohorts, respectively. The predictive parameters for the seven models are shown in Table 3 and Supplementary Table 4.

**Table 3:** Performance indices of the four predictive models for stage I EC in validation cohorts.

	**TJHPEC**	**LR**	**RF**	**GBDT**
**Internal validation cohort (TJH1)**				
AUC (95% CI)	0.9347 (0.9102–0.9592)	0.9149 (0.8859–0.9439)	0.9301 (0.9046–0.9556)	0.9347 (0.9102–0.9592)
Accuracy (95% CI)	91.44% (88.61–94.28%)	89.04% (85.87–92.20%)	89.84% (86.78–92.90%)	90.91% (88.00–93.82%)
Sensitivity (95% CI)	87.30% (79.08–95.52%)	79.37% (69.37–89.36%)	90.48% (83.23–97.72%)	87.30% (79.08–95.52%)
Specificity (95% CI)	92.28% (89.32–95.25%)	91.00% (87.82–94.18%)	89.71% (86.33–93.09%)	91.64% (88.56–94.72%)
PPV (95% CI)	69.62% (59.48–79.76%)	64.10% (53.46–74.75%)	64.04% (54.08–74.01%)	67.90% (57.73–78.07%)
NPV (95% CI)	97.29% (95.43–99.14%)	95.61% (93.27–97.94%)	97.89% (96.23–99.56%)	97.27% (95.40–99.14%)
F1	0.7746	0.7092	0.75	0.7639
Kappa	0.7227	0.6426	0.6886	0.7087
Brier score	0.086	0.11	0.102	0.091
**External validation cohort (RHH)**				
AUC (95% CI)	0.8073 (0.7335–0.8811)	0.8129 (0.7405–0.8853)	0.782 (0.7019–0.8621)	0.7966 (0.7201–0.8731)
Accuracy (95% CI)	82.11% (77.02–87.20%)	78.44% (72.98–83.90%)	77.06% (71.48–82.65%)	81.65% (76.51–86.79%)
Sensitivity (95% CI)	54.17% (34.23–74.10%)	54.17% (34.23–74.10%)	66.67% (47.81–85.53%)	58.33% (38.61–78.06%)
Specificity (95% CI)	85.57% (80.62–90.51%)	81.44% (75.97–86.91%)	78.35% (72.56–84.15%)	84.54% (79.45–89.62%)
PPV (95% CI)	31.71% (17.46–45.95%)	26.53% (14.17–38.89%)	27.59% (16.08–39.09%)	31.82% (18.06–45.58%)
NPV (95% CI)	93.79% (90.23–97.34%)	93.49% (89.77–97.21%)	95.00% (91.62–98.38%)	94.25% (90.79–97.71%)
F1	0.4	0.3562	0.3902	0.4118
Kappa	0.3032	0.2445	0.2778	0.314
Brier score	0.179	0.216	0.229	0.183
**External validation cohort (TJH2)**				
AUC (95% CI)	0.871 (0.8435–0.8985)	0.8577 (0.8284–0.887)	0.8597 (0.8307–0.8887)	0.8685 (0.8407–0.8963)
Accuracy (95% CI)	81.29% (78.40–84.19%)	78.71% (75.66–81.75%)	79.14% (76.12–82.16%)	81.44% (78.55–84.33%)
Sensitivity (95% CI)	82.11% (75.34–88.89%)	81.30% (74.41–88.19%)	86.18% (80.08–92.28%)	82.93% (76.28–89.58%)
Specificity (95% CI)	81.12% (77.91–84.33%)	78.15% (74.76–81.53%)	77.62% (74.21–81.04%)	81.12% (77.91–84.33%)
PPV (95% CI)	48.33% (41.55–55.10%)	44.44% (37.95–50.94%)	45.30% (38.92–51.68%)	48.57% (41.81–55.33%)
NPV (95% CI)	95.47% (93.62–97.32%)	95.11% (93.16–97.06%)	96.31% (94.59–98.03%)	95.67% (93.86–97.48%)
F1	0.6084	0.5747	0.5938	0.6126
Kappa	0.4962	0.4485	0.4711	0.5013
Brier score	0.187	0.213	0.209	0.186

*GBDT, gradient-boosted decision tree; LR, logistic regression; RF, random forest; AUC, area under the receiver-operating characteristic curve; PPV, positive predictive value; NPV, negative predictive value; 95% CI, 95% confidence interval*.

### EC-Related Features in the New Model

**Figure 4:**
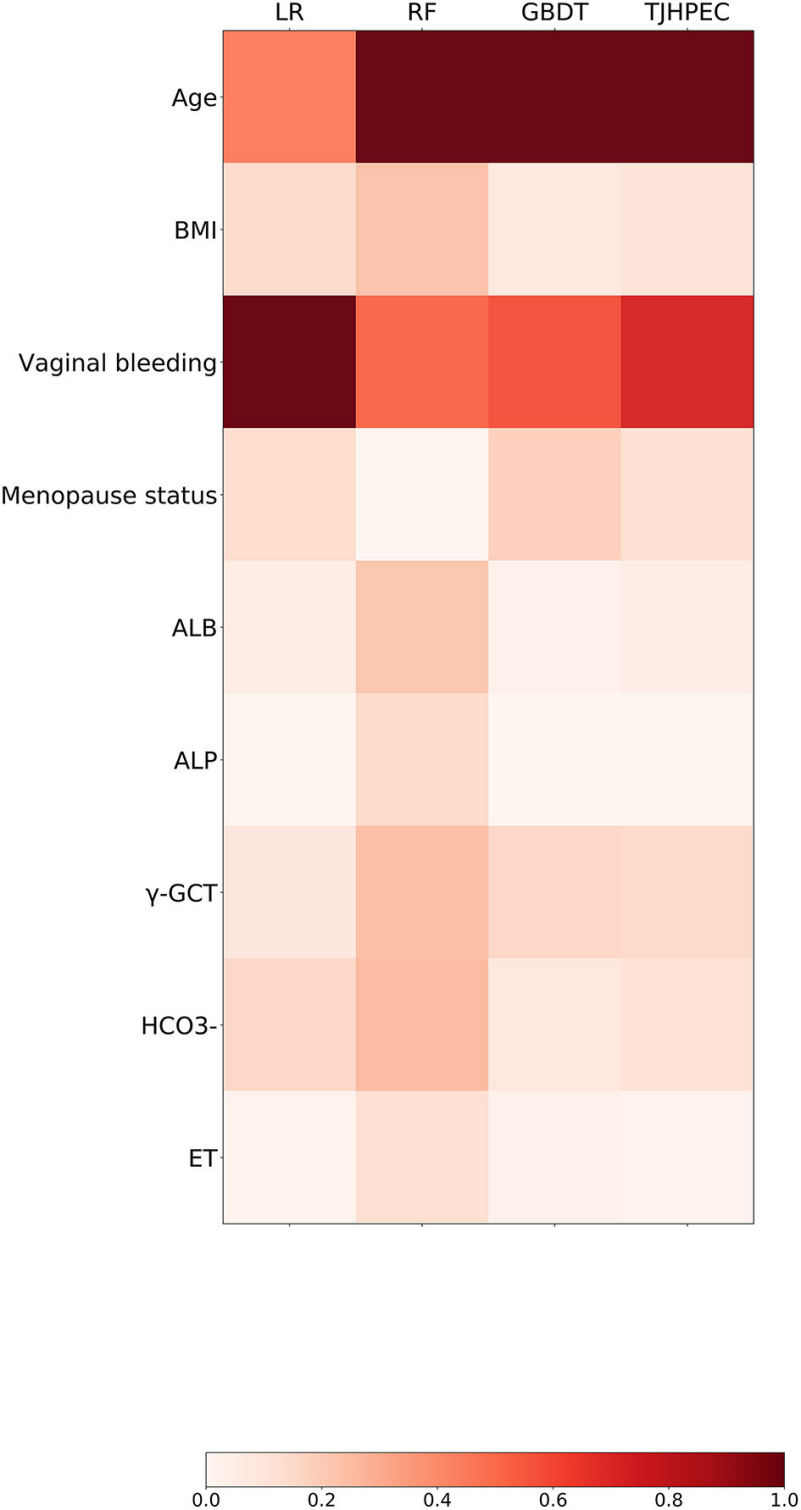
Preponderance ranking of the top 20 features was performed using four models (LR, RF, GBDT, and TJHPEC) for the prediction of EC. The size of each circle represents its relative importance. The depth of the color indicates the importance of each feature in each model.

The relationships between clinical variables and the probability of prediction in the four models are presented in Figure 4. The eight top-ranked features were highly associated with the prediction of EC in the TJHPEC model: age, vaginal bleeding, γ-GGT level, menopause status, concentrations of bicarbonate, albumin, body mass index (BMI) and endometrial thickness (ET) (Supplementary Table 5).

## Discussion

The increasing cancer burden has prompted research on the development of efficient detection tools or models for early diagnosis and prediction of prognosis (13, 14). However, at present, there is no standard EC screening method for the general population. In this retrospective case–control study, we developed an HER- and ML-based computer-assisted ensemble model for diagnosing EC in women in central China. Our analysis showed that the TJHPEC model surpassed the other six ML models for identifying EC in the internal and two external validation cohorts with respective accuracy rates of 91.17, 81.03, and 80.98%, and AUC values of 0.9346, 0.8341, and 0.8649. This new model had a superior prognostic efficacy than the feed-forward artificial NN model described by Pergialiotis et al. (10) who reported a predictive accuracy rate of 85.4% in their internal validation of the model's prediction of the risk of symptomatic postmenopausal women experiencing EC. Moreover, our model had an efficient diagnostic capability for the detection of early EC.

The performance of this new model was slightly worse for the two external validation cohorts than for the internal validation set (Table 2). The TJH2 external validation was a temporal validation that was retrieved from the same hospital as the training and internal validation datasets but at different times. About 40% of patients were missing data for one essential feature (ET) in this dataset vertically, whereas for other features, <20% of patients were missing data; on the horizontal, up to 5 of 9 variables were missing. These may have contributed to the lower predictive ability for the TJH2 cohort although we used predictive mean matching to fill in missing data. The RHH set was a geographic validation. Data for the RHH cohort were from a tertiary hospital, which means that our TJHPEC model may be generalized to other settings. However, data for essential factors such as BMI were also missing for about 40% of patients and the up to 5 variables' data missing also existed, and the patient baseline characteristics were more heterogeneous because of different testing instruments, reagents, and/or reference ranges. The small population may be another reason for the poorer prediction performance in the RHH cohort. Missing data on some variables is inevitable in real-world practice, and our results recommended that all clinical features are needed to take full advantage of this model.

The cohort for training and testing was obtained from 10 years of EHRs relating to the pathology of the endometrium. For some benign disorders, certain characteristics are not part of the routine checkup, which may explain some of the missing data. Although most of the included patients had a rate of missing data of <20%, we still obtained a multidimensional cohort involving features relating to demographics, crucial symptoms and signs, laboratory test results, and tumor-related characteristics shown on ultrasound. Our model currently used nine clinical variables, which are commonly included in the routine physical examinations at most hospitals, makes this new model more practical. The essential risk factors, such as age, vaginal bleeding, menopause status and BMI in our model were also verified in most traditional statistical models, which makes our prediction model more interpretable.

An epidemiological risk model for the prediction of EC was conducted in a large cohort in Western Europe. The risk factors in this model were BMI, menopausal status, ages at menarche and at menopause, oral contraceptive use. The analyses were performed for the total group and by different categories of BMI, parity, age at first full-term pregnancy, duration of menopausal hormone therapy, and smoking status (specific for pre-, peri-, and postmenopausal women). The predicted efficiency over 5 years increased up to 77% compared with the 71% for a model based on age alone (15). Pfeiffer's group developed a predictive model of EC in two large cohorts of US white women aged ≥ 50 years. Their final relative risk model included BMI, menopausal hormone therapy, parity, premenopausal, age at menopause, smoking, and oral contraceptive use, and the AUC was only 0.68 for the validation cohort (16). By comparison, some of the risk factors are not appropriate for evaluation in the Chinese population given the lower prevalence of oral contraceptive and hormone-replacement use than in Europe and the United States (17). Consequently, we did not include these in our model. In addition to demographics and crucial symptoms, our model included laboratory test results and tumor-related characteristics shown on ultrasound, such as γ-GGT, ET, and levels of bicarbonate, albumin, alkaline phosphatase. These have not been included in the other predictive models.

One of the strengths of this study is the wide application scope of TJHPEC. Most previous research involved postmenopausal or older women. Our new TJHPEC model can be used in the general population. Another strength of our research is that our ensemble model TJHPEC had an efficient predictive performance for identifying all cases as well as the early stage of EC.

The main limitation of our research is the retrospective cohort design and the limited training data that were collected from a single institution and produced some missing or unbalanced data. Although we conducted temporal and geographic external validations, the results were not as good as for internal validation because of the small sample sizes, discrepancies in testing instruments, and some missing data. Another limitation is the geographic restriction because the sample population was all from central China. Further prospective studies that include external validation and larger multiracial cohorts are needed to confirm the reliability and to expand the applicable scope of our model.

In conclusion, this study describes a new predictive model of EC in the general population of central China. This model may help clinicians to select more appropriate clinical strategies and to reduce the need for invasive workup and the economic burden. In the future, a larger prospective cohort study from multiple centers will be conducted to validate and warrant the model's predictive efficiency.

## Data Availability Statement

The original contributions presented in the study are included in the article/Supplementary Material, further inquiries can be directed to the corresponding author/s.

## Ethics Statement

The studies involving human participants were reviewed and approved by the Medical Ethics Committee of Tongji Hospital Affiliated to Tongji Medical College of Huazhong University of Science and Technology. Written informed consent for participation was not required for this study in accordance with the national legislation and the institutional requirements.

## Author Contributions

WW, WS, and SW: conception and design. WW, SY, and YX: provision of study material or patients. SY, YX, and QZ: collection and/or assembly of data. SY, YX, and XZ: data analysis and interpretation. WW, SY, YX, WS, and SW: manuscript writing. WW, YX, SY, QZ, XZ, ZL, WS, and SW: final approval of manuscript. All authors contributed to the article and approved the submitted version.

## Funding

This study was supported by grants from the National Natural Science Foundation of China (No. 81701420), the Science and Technology Commission Shanghai Municipality (STCSM) (No. 19511121002), and the Shanghai Engineering Research Center of Intelligent Computing System (No. 19DZ2252600).

## Conflict of Interest

The authors declare that the research was conducted in the absence of any commercial or financial relationships that could be construed as a potential conflict of interest.

## Publisher's Note

All claims expressed in this article are solely those of the authors and do not necessarily represent those of their affiliated organizations, or those of the publisher, the editors and the reviewers. Any product that may be evaluated in this article, or claim that may be made by its manufacturer, is not guaranteed or endorsed by the publisher.
