# Supplementary material for: Prediction of Endometrial Carcinoma Using the Combination of Electronic Health Records and an Ensemble Machine Learning Method
Source: Front Med (Lausanne). 2022 Mar 4;9:851890. doi: 10.3389/fmed.2022.851890 (PMC8931475; doi:10.3389/fmed.2022.851890)
Supplement: Supplementary file 1 [file Data_Sheet_1.docx]

Supplementary table 1. Pathological diagnosis of the total cohort.

| Pathological diagnosis | Number |
| --- | --- |
| Endometrial carcinoma | 551 |
| Adenocarcinoma | 515 |
| Clear cell carcinoma | 6 |
| Serous carcinoma | 17 |
| Undifferentiated carcinoma | 10 |
| Carcinosarcoma | 1 |
| Large or small cell neuroendocrine carcinoma | 2 |
| Benign endometrial disorder | 2318 |
| Normal endometrium | 1610 |
| polyps | 651 |
| Hyperplasia without atypia | 57 |

Supplementary table 2. Demographic characteristics in validation cohorts.

| **Characteristic**  Mean±SD or N (%) | **TJH1 cohort** | | **RHH cohort** | | **TJH2 cohort** | |
| --- | --- | --- | --- | --- | --- | --- |
|  | **Benign (n=311)** | **EC (n=74)** | **Benign (n=194)** | **EC (n=38)** | **Benign (n=572)** | **EC (n=143)** |
| Menstrual history |  |  |  |  |  |  |
| Menarche age | 14 ± 1 | 14 ± 2 | 14 ± 2 | 14 ± 1 | 14 ± 8 | 14 ± 2 |
| Menstrual period | 6 ± 5 | 6 ± 4 | 6 ± 2 | 5 ± 1 | 6 ± 2 | 6 ± 2 |
| Menorrhagia | 26 (8.4) | 5 (6.8) | 15 (7.7) | 4 (10.5) | 46 (8) | 11 (7.7) |
| Dysmenorrhea | 52 (16.7) | 6 (8.1) | 38 (19.6) | 12 (31.6) | 135 (23.6) | 18 (12.6) |
| Symptoms |  |  |  |  |  |  |
| Irregular menstrual cycle | 20 (6.4) | 2 (2.7) | 24 (12.4) | 5 (13.2) | 63 (11) | 33 (23.1) |
| Family history | 34(10.9) | 8 (10.8) | 0 | 2 (5.3) | 41 (7.2) | 12 (8.4) |
| Comorbidity |  |  |  |  |  |  |
| Diabetes | 9 (2.9) | 5 (6.8) | 2 (1) | 5 (13.2) | 16 (2.8) | 9 (6.4) |
| Hpertension | 16 (5.1) | 13 (17.6) | 2 (1) | 13 (34.2) | 27 (4.7) | 25 (18) |
| Laboratory test |  |  |  |  |  |  |
| Lymphocytes, 10^^9^/L | 1.76 ± 0.60 | 1.77 ± 0.66 | 1.69 ± 0.56 | 1.63 ± 0.48 | 1.80 ± 0.57 | 1.80 ± 0.64 |
| Lymphocyte, % | 31.9 ± 10.5 | 30.6 ± 10.3 | 33.4 ± 20.1 | 30.4 ± 8.9 | 31.3 ± 9.4 | 30.9 ± 9.6 |
| Eosinophils, % | 1.9 ± 1.8 | 1.6 ± 1.1 | 2.2 ± 2.1 | 2.5 ± 1.7 | 2.0 ± 1.8 | 2.1 ± 1.8 |
| Monocytes, 10^9/L | 0.41 ± 0.15 | 0.42 ± 0.19 | 0.41 ± 0.53 | 0.39 ± 0.19 | 0.44 ± 0.18 | 0.45 ± 0.19 |
| Basophils, 10^9/L | 0.02 ± 0.03 | 0.02 ± 0.01 | 0.02 ± 0.02 | 0.02 ± 0.02 | 0.03 ± 0.02 | 0.03 ± 0.02 |
| HCT, % | 36.7 ± 4.7 | 36.0 ± 5.0 | 35.7 ± 4.6 | 33.7 ± 6.7 | 35.6 ± 5.2 | 36.2 ± 5.3 |
| MCV, fL | 86.16 ± 7.55 | 86.99 ± 6.30 | 87.58 ± 10.15 | 86.94 ± 9.79 | 85.9 ± 8.0 | 87.4 ± 7.6 |
| MCHC, g/L | 326 ± 19 | 327 ± 16 | 316 ± 34 | 325 ± 20 | 326 ± 20 | 330 ± 16 |
| Platelets, 10^9/L | 239 ± 67 | 228 ± 72 | 209 ± 69 | 210 ± 66 | 254 ± 76 | 243 ± 76 |
| AST, U/L | 18 ± 15 | 22 ± 11 | 21 ± 9 | 23 ± 9 | 19 ± 14 | 21 ± 12 |
| TC, mmol/l | 3.99 ± 0.77 | 4.36 ± 1.01 | 4.83 ± 1.29 | 4.77 ± 1.02 | 4.16 ± 0.85 | 4.41 ± 0.88 |
| TP, g/L | 70.3 ± 6.7 | 67.7 ± 7.8 | 70.3 ± 9.0 | 68.9 ± 7.1 | 72.4 ± 6.3 | 70.7 ± 5.4 |
| Neutrophils, % | 57.1 ± 10.8 | 61.6 ± 11.3 | 58.7 ± 9.6 | 59.4 ± 11.3 | 58.9 ± 10.8 | 59.1 ± 10.9 |
| CA-125, U/ml | 37.80 ± 55.63 | 35.56 ± 53.37 | 48.43 ± 143.40 | 57.99 ± 138.85 | 47.39 ± 62.06 | 102.51 ± 491.28 |
| TBIL, μmol/L | 10.4 ± 5.0 | 9.5 ± 4.3 | 12.8 ± 7.2 | 12.0 ± 4.4 | 8.9 ± 7.1 | 8.6 ± 4.6 |
| DBIL, μmol/L | 3.0 ± 1.5 | 2.6 ± 1.26 | 4.5 ± 7.1 | 3.6 ± 1.5 | 3.5 ± 5.4 | 3.3 ± 1.5 |
| BUN, mmol/L | 4.20 ± 1.28 | 4.75 ± 1.48 | 20.74 ± 220.30 | 4.92 ± 1.50 | 4.39 ± 1.52 | 5.39 ± 3.03 |
| UA, μmol/L | 246.2 ± 63.3 | 248.6 ± 73.8 | 230.8 ± 66.6 | 266.2 ± 96.5 | 270.3 ± 66.6 | 307.1 ± 94.8 |
| Creatinine, μmol/L | 55 ± 9 | 58 ± 11 | 57 ± 12 | 59 ± 10 | 58 ± 41 | 74 ± 111 |
| eGFR, ml/min/1.73m^2^ | 113.1 ± 19.6 | 97.0 ± 17.0 | 79.2 ± 19.6 | 70.3 ± 12.9 | 110.6 ± 16.1 | 95.1 ± 20.9 |

Abbreviations: TBIL, total bilirubin; ALP, alkaline phosphatase; BUN, blood urea nitrogen; γ-GGT, γ-glutamyl-transpeptidase; TC, total cholesterol; AST, aspartate aminotransferase; UA, uric acid; Diabetes, type II diabetes; Family history, Family history of malignant diseases; MCV, mean corpuscular volume; HCT, hematocrit; eGFR, estimated glomerular filtration rate; MCHC, mean corpuscular hemoglobin concentration; TP, total protein; DBIL, direct bilirubin; ALB, albumin; HCO3-, concentrations of bicarbonate; N/A, none available.

Supplementary table 3. Performance indices of other three predictive models (SVM, NN, KNN) for total EC in validation cohorts.

|  | **SVM** | **NN** | **KNN** |
| --- | --- | --- | --- |
| **Internal validation cohort (TJH1)** | | | |
| AUC  (95% CI) | 0.9174  (0.8899-0.9449) | 0.8916  (0.859-0.9242) | 0.8729  (0.8369-0.9089) |
| Accuracy  (95% CI) | 89.09%  (85.98–92.20%) | 84.94%  (81.36–88.51%) | 85.19%  (81.65–88.74%) |
| Sensitivity  (95% CI) | 78.38%  (69.00–87.76%) | 78.38%  (69.00–87.76%) | 78.38%  (69.00–87.76%) |
| Specificity  (95% CI) | 91.64%  (88.56–94.72%) | 86.50%  (82.70–90.29%) | 86.82%  (83.06–90.58%) |
| PPV  (95% CI) | 69.05%  (59.16–78.93%) | 58.00%  (48.33–67.67%) | 58.59%  (48.88–68.29%) |
| NPV  (95% CI) | 94.68%  (92.15–97.22%) | 94.39%  (91.71–97.06%) | 94.41%  (91.74–97.07%) |
| F1 | 0.7342 | 0.6667 | 0.6705 |
| Kappa | 0.6659 | 0.5721 | 0.5776 |
| Brier | 0.109 | 0.151 | 0.148 |
| **External validation cohort (RHH)** | | | |
| AUC  (95% CI) | 0.8043  (0.7409-0.8677) | 0.8195  (0.7592-0.8798) | 0.7568  (0.6844-0.8292) |
| Accuracy  (95% CI) | 80.17%  (75.04–85.30%) | 75.43%  (69.89–80.97%) | 74.14%  (68.50–79.77%) |
| Sensitivity  (95% CI) | 63.16%  (47.82–78.49%) | 71.05%  (56.63–85.47%) | 65.79%  (50.71–80.87%) |
| Specificity  (95% CI) | 83.51%  (78.28–88.73%) | 76.29%  (70.30–82.27%) | 75.77%  (69.74–81.80%) |
| PPV  (95% CI) | 42.86%  (29.90–55.82%) | 36.99%  (25.91–48.06%) | 34.72%  (23.73–45.72%) |
| NPV  (95% CI) | 92.05%  (88.05–96.04%) | 93.08%  (89.14–97.03%) | 91.88%  (87.64–96.11%) |
| F1 | 0.5106 | 0.4865 | 0.4545 |
| Kappa | 0.392 | 0.3455 | 0.3057 |
| Brier | 0.198 | 0.246 | 0.259 |
| **External validation cohort (TJH2)** | | | |
| AUC  (95% CI) | 0.8337  (0.8028-0.8646) | 0.8269  (0.7952-0.8586) | 0.7892  (0.7535-0.8249) |
| Accuracy  (95% CI) | 80.98%  (78.10–83.86%) | 76.22%  (73.10–79.34%) | 76.08%  (72.96–79.21%) |
| Sensitivity  (95% CI) | 76.92%  (70.02–83.83%) | 78.32%  (71.57–85.08%) | 73.43%  (66.19–80.67%) |
| Specificity  (95% CI) | 81.99%  (78.84–85.14%) | 75.70%  (72.18–79.21%) | 76.75%  (73.29–80.21%) |
| PPV  (95% CI) | 51.64%  (44.93–58.35%) | 44.62%  (38.47–50.77%) | 44.12%  (37.81–50.43%) |
| NPV  (95% CI) | 93.43%  (91.26–95.59%) | 93.32%  (91.05–95.59%) | 92.03%  (89.60–94.46%) |
| F1 | 0.618 | 0.5685 | 0.5512 |
| Kappa | 0.4978 | 0.421 | 0.4017 |
| Brier | 0.19 | 0.238 | 0.239 |

Abbreviations: SVM, support vector machine; KNN, support vector machine; NN, neural network; AUC, area under the receiver operating characteristics curve; PPV, positive predictive value; NPV, negative predictive value; 95% CI, 95% confidence interval.

Supplementary table 4. Performance indices of other three predictive models (SVM, NN, KNN) for stage I EC in validation cohorts.

|  | **SVM** | **NN** | **KNN** |
| --- | --- | --- | --- |
| **Internal validation cohort (TJH1)** | | | |
| AUC  (95% CI) | 0.9131  (0.8837-0.9425) | 0.883  (0.8474-0.9186) | 0.8701  (0.832-0.9082) |
| Accuracy  (95% CI) | 89.30%  (86.17–92.44%) | 84.76%  (81.12–88.40%) | 85.03%  (81.41–88.64%) |
| Sensitivity  (95% CI) | 77.78%  (67.51–88.04%) | 76.19%  (65.67–86.71%) | 76.19%  (65.67–86.71%) |
| Specificity  (95% CI) | 91.64%  (88.56–94.72%) | 86.50%  (82.70–90.29%) | 86.82%  (83.06–90.58%) |
| PPV  (95% CI) | 65.33%  (54.56–76.10%) | 53.33%  (43.03–63.64%) | 53.93%  (43.58–64.29%) |
| NPV  (95% CI) | 95.32%  (92.92–97.71%) | 94.72%  (92.12–97.32%) | 94.74%  (92.14–97.33%) |
| F1 | 0.7101 | 0.6275 | 0.6316 |
| Kappa | 0.6452 | 0.5354 | 0.541 |
| Brier | 0.107 | 0.152 | 0.15 |
| **External validation cohort (RHH)** | | | |
| AUC  (95% CI) | 0.7521  (0.6651-0.8391) | 0.7952  (0.7183-0.8721) | 0.712  (0.6167-0.8073) |
| Accuracy  (95% CI) | 80.73%  (75.50–85.97%) | 75.23%  (69.50–80.96%) | 73.85%  (68.02–79.69%) |
| Sensitivity  (95% CI) | 58.33%  (38.61–78.06%) | 66.67%  (47.81–85.53%) | 58.33%  (38.61–78.06%) |
| Specificity  (95% CI) | 83.51%  (78.28–88.73%) | 76.29%  (70.30–82.27%) | 75.77%  (69.74–81.80%) |
| PPV  (95% CI) | 30.43%  (17.14–43.73%) | 25.81%  (14.91–36.70%) | 22.95%  (12.40–33.50%) |
| NPV  (95% CI) | 94.19%  (90.69–97.68%) | 94.87%  (91.41–98.33%) | 93.63%  (89.81–97.45%) |
| F1 | 0.4 | 0.3721 | 0.3294 |
| Kappa | 0.2985 | 0.2536 | 0.2036 |
| Brier | 0.193 | 0.248 | 0.261 |
| **External validation cohort (TJH2)** | | | |
| AUC  (95% CI) | 0.835  (0.8029-0.8671) | 0.8288  (0.7959-0.8617) | 0.7905  (0.7532-0.8278) |
| Accuracy  (95% CI) | 81.29%  (78.40–84.19%) | 76.12%  (72.95–79.29%) | 76.40%  (73.25–79.56%) |
| Sensitivity  (95% CI) | 78.05%  (70.73–85.36%) | 78.05%  (70.73–85.36%) | 74.80%  (67.12–82.47%) |
| Specificity  (95% CI) | 81.99%  (78.84–85.14%) | 75.70%  (72.18–79.21%) | 76.75%  (73.29–80.21%) |
| PPV  (95% CI) | 48.24%  (41.30–55.18%) | 40.85%  (34.57–47.14%) | 40.89%  (34.47–47.31%) |
| NPV  (95% CI) | 94.56%  (92.56–96.55%) | 94.13%  (91.98–96.28%) | 93.40%  (91.16–95.65%) |
| F1 | 0.5963 | 0.5363 | 0.5287 |
| Kappa | 0.4832 | 0.396 | 0.3889 |
| Brier | 0.187 | 0.239 | 0.236 |

Abbreviations: SVM, support vector machine; KNN, support vector machine; NN, neural network; AUC, area under the receiver operating characteristics curve; PPV, positive predictive value; NPV, negative predictive value; 95% CI, 95% confidence interval.

Supplementary table 5. Rank of all features in four models for exploring high risk of EC.

|  | **LR** | **RF** | **GBDT** | **TJHPEC** |
| --- | --- | --- | --- | --- |
| Age | 0.4469 | 1 | 1 | 1 |
| BMI | 0.1426 | 0.2291 | 0.0662 | 0.0986 |
| Vaginal bleeding | 1 | 0.5137 | 0.5676 | 0.7139 |
| Menopause status | 0.1332 | 0 | 0.1762 | 0.1199 |
| ALB | 0.0541 | 0.2149 | 0.0208 | 0.0431 |
| ALP | 0 | 0.1464 | 0 | 0 |
| γ-GGT | 0.0808 | 0.2396 | 0.1538 | 0.1478 |
| HCO3- | 0.1546 | 0.2505 | 0.0757 | 0.1129 |
| ET | 0.015 | 0.1224 | 0.0196 | 0.0116 |

A normalized probability of risk for endometrial carcinoma range from 0 to 1 was produced by each model.

Abbreviations: BMI, body mass index; ALB, albumin; ALP, alkaline phosphatase; γ-GGT, γ-glutamyl-transpeptidase; HCO3-, concentrations of bicarbonate; ET, Endometrial Thickness.

Supplementary table 6. The definition of the predictors as follows:

| Vaginal bleeding | bleeding from uterine corpus other than due to pregnancy |
| --- | --- |
| Menopause status | menopausal |
| Irregular cycle | the length of menstrual cycle is ＜21 days or ＞35 days |
| Family history | have previous malignant diseases |
| Menstrual period | the length of the period |
| Parity | the number of births |
| Menorrhagia | determined by patient, which is referred as the bleeding volume ＞80 ml. |
