# Supplementary material for: Prediction of Endometrial Carcinoma Using the Combination of Electronic Health Records and an Ensemble Machine Learning Method
Source: Front Med (Lausanne). 2022 Mar 4;9:851890. doi: 10.3389/fmed.2022.851890 (PMC8931475; doi:10.3389/fmed.2022.851890)
Supplement: Supplementary file 2 [file Data_Sheet_2.docx]

Supplementary figure 1.


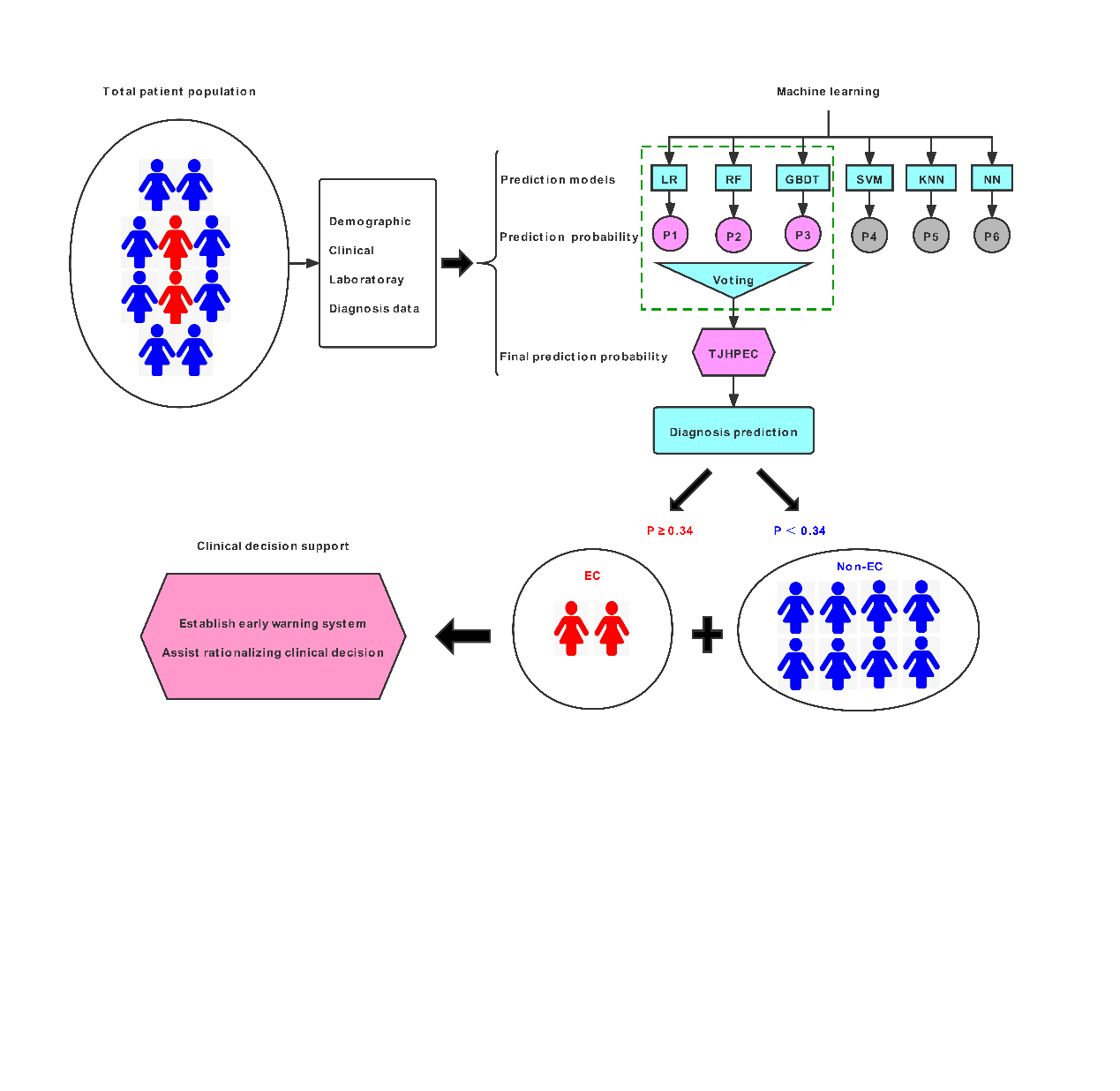


Schematic diagram of our TJHPEC predictive model. Six machine learning models (LR, RF, GBDT, SVM, KNN, and NN) were adopted based on the included patients’ demographic, clinical, and laboratory features and used to predict the risk of endometrial carcinoma (EC). The top three optimal diagnostic–predictive models (RF, GBDT, and LR) were fused to develop a new ensemble model named TJHPEC by voting (See Methods). TJHPEC yielded a diagnostic risk probability range from 0 to 1 and a decision-making threshold of 0.34. Patients with a probability of <0.34 were allotted to the non-EC group and those with a higher probability to the EC group. Abbreviations: TJHPEC, Tongji Hospital prediction of endometrial cancer; LR, logistic regression; SVM, support vector machine; GBDT, gradient boosted decision tree; NN, neural network; EC, endometrial carcinoma.

Supplementary figure 2.


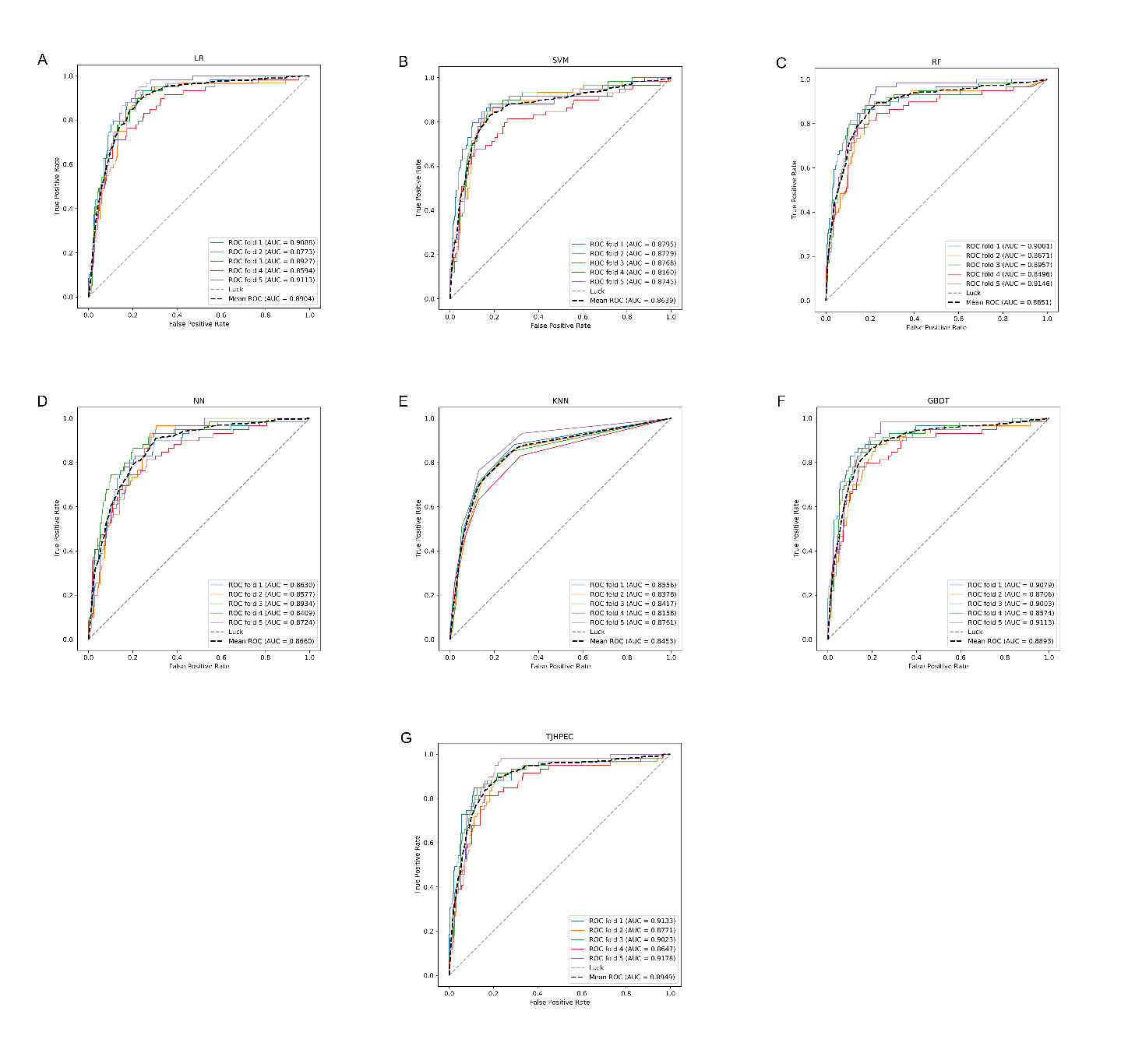


Fivefold cross-validation of the seven predictive models (LR, SVM, RF, NN, KNN, GBDT and TJHPEC).
